# Treatment alternatives for dry mouth: A scoping review

**DOI:** 10.4317/jced.59912

**Published:** 2022-10-01

**Authors:** Sven E. Niklander, Matías Martínez, Andrea Miranda, Macarena Rodriguez

**Affiliations:** 1 Unidad de Patología y Medicina Oral, Facultad de Odontologia, Universidad Andres Bello, Viña del Mar, Chile; 2 Departamento de Morfología, Facultad de Medicina, Universidad Andres Bello, Viña del Mar, Chile

## Abstract

**Background:**

Saliva is a biological fluid essential for the maintenance of a proper oral health. Its absence predisposes to differences pathologies, including dental caries, fungal infections among many others, significantly affecting the oral health related quality of life (OHRQoL). There is a large variety of treatment alternatives available for dry mouth, which increases constantly. Objective: To identify new treatment alternatives for dry mouth.

**Material and Methods:**

We conducted a systematic search in PubMed/MEDLINE, Web of Science, Scopus and Ebsco. Articles published between January 2015 and January 2020 were retrieved and reviewed by two independent evaluators.

**Results:**

Nineteen studies met the inclusion criteria and were included for analysis. Local therapies were the most evaluated agents, followed by systemic and non-conventional treatments. Most local therapies showed certain utility for the management of dry mouth and the improvement of OHRQoL. These formulations were mainly based on natural agents, including malic acid, thyme honey, ginger, among others.

**Conclusions:**

Local agents are first line treatment alternatives for dry mouth sensation, with a reported efficiency that varies between studies, and with a low number of reported adverse side-effects. Nevertheless, care must be taken when interpreting these results, as is difficult to compare studies within each other due large heterogeneity in study design and outcomes being measured.

** Key words:**Xerostomia, dry mouth, hyposalivation, saliva, mouth dryness.

## Introduction

Dry mouth sensation or xerostomia is the subjective feeling of dry mouth which can or not be accompanied by hyposalivation. The term hyposalivation refers to an objective, measurable decrease of the salivary output. Importantly, these terms are not synonyms, as not all patients with xerostomia will have a reduced salivary flow, and not every patient with hyposalivation will complain of dry mouth (1). Thus, they should not be used interchangeably. There are many causes for experiencing dry mouth. In many cases, xerostomia is caused by salivary gland hypofunction due to an autoimmune disease such as Sjögren’s syndrome or scleroderma (2), or due to irradiation to the head and neck as part of cancer therapy (3). But in other occasions, xerostomia is observed in patients with normal salivary output frequently associated depression, anxiety or as an adverse side-effect of medication (probably the most common cause of oral dryness) (1).

Experiencing dry mouth can have serious consequences for the oral health. It has been associated with difficulty in speaking and/or swallowing, dental erosions, predisposition to dental caries, fungal and bacterial infections, burning sensation, lack of denture retention, taste disturbance and halitosis (4), affecting negatively the OHRQoL (1,5,6) with significant impact on physical, emotional and social aspects; even more than dental caries (7).

The treatment of xerostomia is complex as it mainly depends on its cause. On many occasions, it is solely based on alleviating patients’ symptoms as the underlying cause (e.g., Sjögren’s syndrome, radiation therapy) has produced irreversible damage to the salivary glands that cannot be corrected (1). Commonly, dry mouth treatment is based on the use of local agents as these offer symptom alleviation with minor adverse-side effects. Local agents are intended either to stimulate the salivary glands to increase salivary flow (salivary stimulants) or to replace the absence of saliva (salivary substitutes) (8). Systemic sialogogues, such as pilocarpine or cevimeline are also an alternative, but these are not available worldwide and have significant adverse-side effects, thus their use is more limited (9,10). During the last years, other non-conventional treatment alternatives have emerged, group known as alternative therapies, which include: electric stimulation (11), acupuncture (12) and genetic therapy (11).

Due to the increasing report of new agents for the treatment of dry mouth, the aim of this scoping review was to identify new treatment alternatives for xerostomia reported during the last years with potential to be used in clinical practice.

## Material and Methods

-Study design

This scoping review was performed according to the Preferred Reporting Items for Systematic Reviews and Meta-Analysis (PRISMA) guidelines and scoping review guidelines from Joanna Briggs Institute (13). The research questions was based on the PICOT format (14) as follows: Population (P): patients with xerostomia. Intervention (I): any intervention for dry mouth. Comparison (C): no restrictions. Outcome: alleviation of dry mouth symptoms and/or increase in salivary output. Type of study (T): Randomized controlled trials (RCT).

-Data sources and search strategies

An electronic search was performed using Pubmed/MEDLINE, EBSCO, Scopus and Web of Science databases, comprising a period between January 2015 and January 2020, using the following key words: “Xerostomia”[Mesh] AND (“Therapeutics/drug therapy”[Mesh] OR “Therapeutics/drug effects”[Mesh] OR “Therapeutics/pharmacology”[Mesh]), Xerostomia AND (treatment OR therapy) AND Clinical trial, y and “Xerostomia AND therapy AND Clinical trial”.

-Inclusion criteria

Selected articles were considered eligible when they fulfilled the following criteria: 1) Randomized control trials (RCTs) assessing treatment alternatives for xerostomia and/or hyposalivation; 2) articles pubslihed between January 2015 and January 2020; 3) full text articles in English.

-Exclusion criteria

Exclusion criteria were the following: 1) *in vitro* or animal studies; 2) studies assessing treatments to prevent the development of dry mouth; 3) review articles, book chapters, systematic reviews, meta-analysis, case reports, cross-sectional studies, or case series.

-Data extraction

After article selection, these were reviewed by two independent evaluators and were considered eligible if they accomplished the inclusion criteria. Disagreements were discussed between the evaluators. All the extracted data was registered in a specifically design spreadsheet to eliminate possible mistakes.

## Results

**Figure 1:**
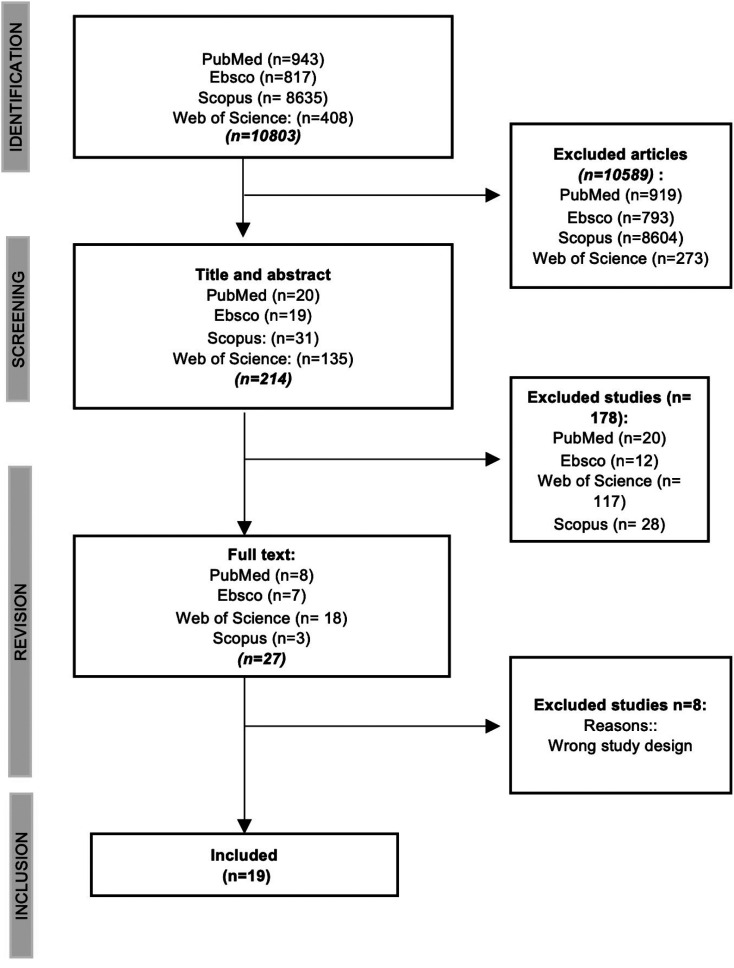
Prisma flowchart.

The search identified 10.803 articles, from which 10589 were excluded after duplicate removal and filtration by date of publication. Thus, 214 studies had their title and abstract reviewed, of which 178 were excluded. Full text articles were obtained from 27 studies. Of these, 8 articles were excluded because they did not meet the inclusion criteria, therefore, 19 articles were included in this study for analysis (Fig. 1).

Head and Neck radiotherapy was the most common cause of xerostomia in all studies (42.11%), followed by primary Sögren’s syndrome (15.8%). A large variety of instruments were used for establishing the diagnosis of xerostomia. The visual analogue scale (VAS) was the most common diagnostic method, followed by the Common Terminology Criteria for Adverse Events (CTCAE) scale, Xerostomia Questionnaire (XQ), Xerostomia Inventory (XI), among others (Table 1,  Table 1 cont.). Therapies were classified according to their application method into three categories: local therapies, systemic therapies, or alternative therapies. Of the 19 studies included in this review, 13 (68.4%) assessed the effect of local therapies, 3 (18.8%) used different vegetal products administered systemically whether in capsules or infusions, and 3 (18.8%) studied the effect of non-conventional therapies such as transcutaneous stimulation or laser therapy. The follow-up period ranged from 8 days (15) to 4 years (16), but in most of the studies (63.1%), the follow-up period was not greater than 4 weeks, and only in 10.5% was greater than 12 months (Table 1,  Table 1 cont.).

**Table 1:**
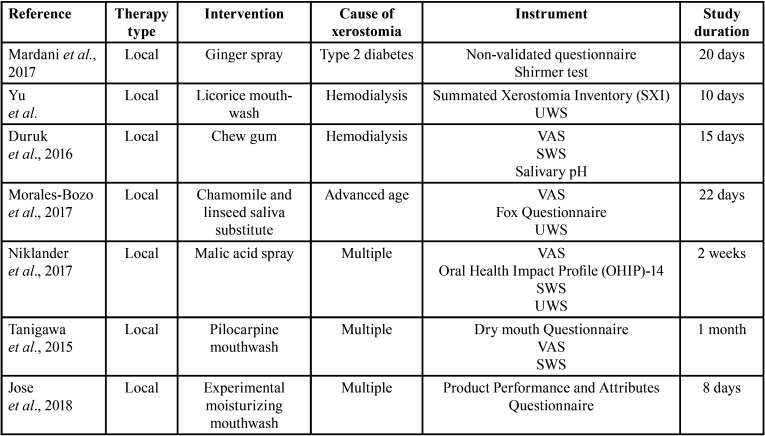
Details of the included studies.

**Table 1 cont.**
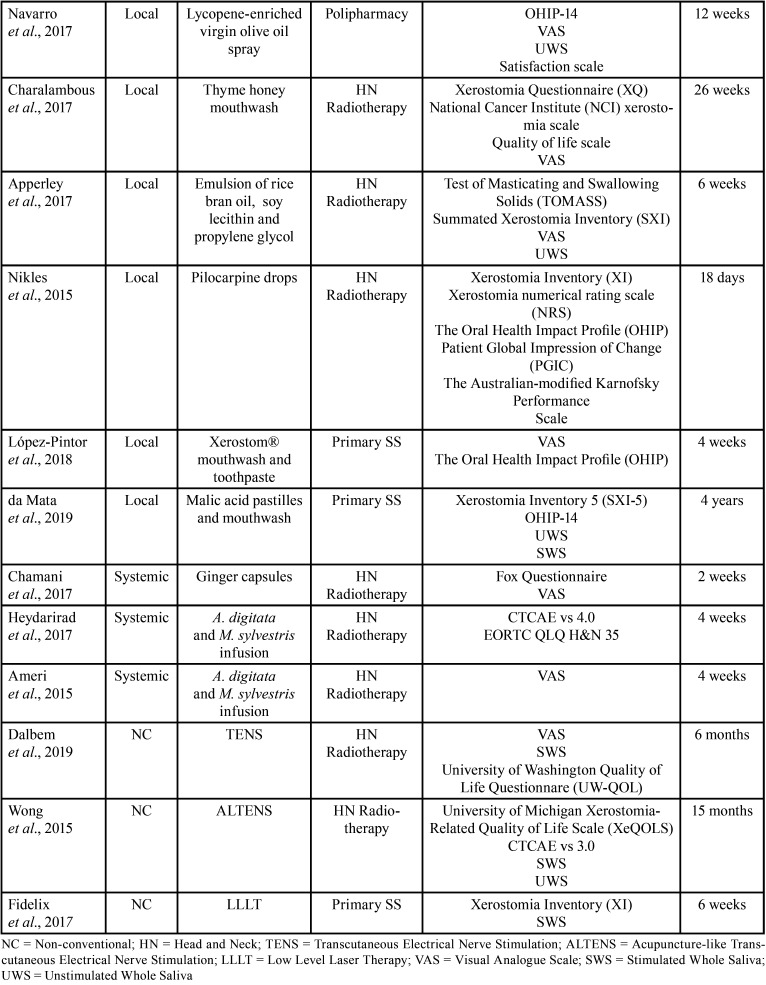
Details of the included studies.

-Local therapies 

Thirteen studies assessed local therapies for the treatment of dry mouth including a variety of application methods, such as: mouthwashes, lozenges, sprays, salivary substitutes, droplets, and chew gum (Tables  Table 1, Table 2,  Table 2 cont.).

**Table 2:**
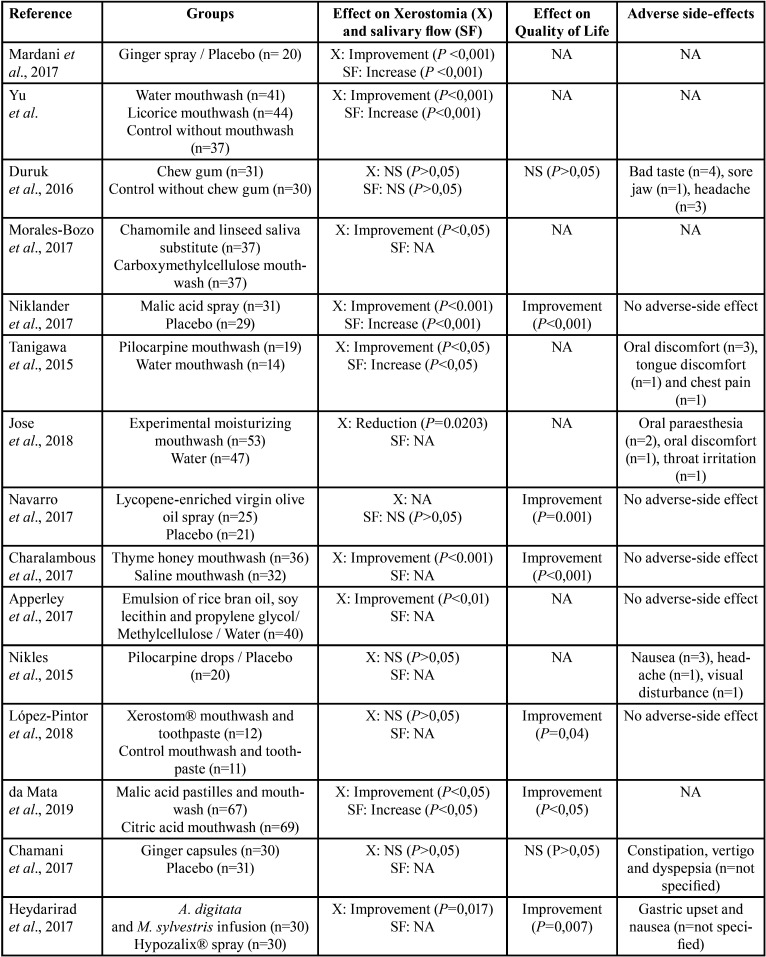
Summary of the main results of the included studies.

**Table 2 cont.**
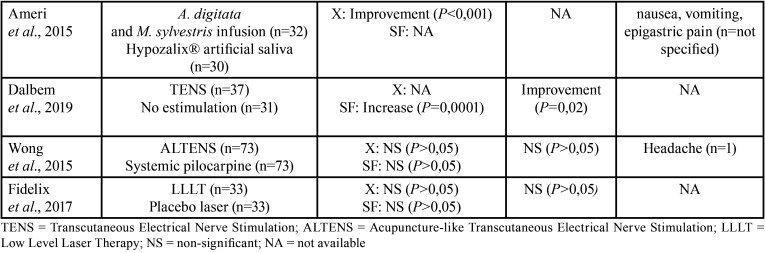
Summary of the main results of the included studies.

Six studies evaluated different compounds as mouthwashes; 4 studies compared a treatment mouthwash with a placebo mouthwash, 1 study compared a treatment mouthwash with another treatment agent with different application method (lozenge) and 1 study compared the application of a treatment mouthwash in combination with a treatment toothpaste with placebos. In general, all mouthwashes reported some favourable results with no or reduced adverse side effects. Pilocarpine (17) and licorice mouthwashes (18) were shown to improve dry mouth sensation and salivary flow rates when compared to placebo mouthwashes. Similarly, a mouthwash based on thyme honey significantly improved dry mouth sensation and OHRQoL in patients with drug-induced xerostomia (19). A study assessing a newly developed experimental mouthwash based on glycerine, xylitol, sorbitol, and propylene glycol, among other components (15), found a significant relieve in dry mouth sensation when compared to water, but only in patients suffering from dry mouth associated to Sjögren’s syndrome. A randomized clinical trial that compared a malic acid lozenge with a citric acid mouthwash in their ability to improve dry mouth sensation and OHRQoL in Sjögren’s syndrome patients, reported both gustatory salivary stimulants to improve dry mouth sensation, OHRQoL and salivary output, but with a greater effect in the malic acid lozenge group (16). Finally, another randomized controlled trial that assessed the combined application of Xerostom mouthwash and Xerostom toothpaste in primary Sjögren’s syndrome patients, reported a significant improvement in OHRQoL before and after treatment, which was not observed in the control group. No differences in the intragroup VAS scale before and after treatment were observed (20) (Table 2, 2 cont.).

Three clinical trials assessed the use of different agents as sprays, including olive oil with lycopene (21), malic acid (6) and ginger (22) sprays. Both the olive oil with lycopene (21) and malic acid (6) sprays reported an improvement in OHRQoL, but only the 1% malic acid spray significantly improved dry mouth sensation and salivary flow rate (6). The ginger herbal spray also improved both xerostomia and salivary flow (22).

Two studies evaluated the use of natural emulsions as saliva substitutes. Morales-Bozo *et al*. assessed a combined infusion of chamoline and linseed, reporting a significant improvement in dry mouth symptoms, thick saliva sensation and difficulty in swallowing, compared to a conventional saliva substitute (23). Apperley *et al*. evaluated a saliva substitute composed of rice bran oil and soy lecithin but failed to demonstrate any clinical benefit over other conventional treatments for xerostomia (24). One study tested the effect of pilocarpine drops for the alleviation of dry mouth symptoms in advanced cancer patients, but found no benefit, with significant adverse-side effect (25). Finally, a randomized clinical trial assessed the effect of chewing gum during haemodialysis , but found no effect on the salivary output or dry mouth symptoms (26).

-Systemic therapies

Systemic agents were evaluated by 3 studies. Ginger capsules swallowed three times a day over a period of 14 days, did not significantly improved xerostomia nor OHRQoL in patients with post-radiotherapy xerostomia, when compared to a control group (27). A herbal infusion containing Malva sylvestris and Alcea digitata was reported to be effective improving dry mouth sensation (28,29) and OHRQol (29) by two randomized-controlled trials (Table 2,  Table 2 cont.).

-Non-conventional treatments

This category refers to treatments different than systemic or local agents, which included the use of transcutaneous stimulation (30,31) and low-level laser therapy (LLLT) (32). Wong *et al*. assessed the use of acupuncture-like transcutaneous electrical stimulation (ALTENS) for the treatment of radiation-induced xerostomia and reported no changes in salivary flow nor OHRQoL after 24 sessions of 20 minutes each (31). Dalbem *et al*. assessed transcutaneous stimulation (TENS) for the treatment of radiation-induced xerostomia. TENS was applied bilaterally on submandibular and parotid glands, 20 minutes per sessions, twice a week, for 4 weeks. The authors reported a significant increase in salivary flow rate and OHRQoL (30). Fidelix *et al*. studied the effect of LLLT in the treatment of xerostomia in Sjögren’s syndrome, reporting no improvement in dry mouth symptoms nor salivary flow rates after 12 treatment sessions over a period of 6 weeks (32).

## Discussion

Dry mouth is a common clinical condition with different possible causes. Dry mouth treatment is usually focused whether on replacing missing saliva with a solution that mimics saliva or its main properties, or by stimulating the salivary glands (or their remaining’s) to increase salivary output. For either approach, a large variety of reports assessing different treatments modalities exists, which increases every year in an exponential way. Thus, it can be very difficult for clinicians to be updated with emerging treatment modalities. Therefore, the aim of this scoping review was to identify new treatment alternatives for xerostomia reported during the last years with potential to be used in clinical practice.

Of the 19 studies included in this review, most of them (68.4%), assessed the use of local treatments. Many authors consider local therapies as first line treatments because their application is simpler for patients and is associated with no or low number of adverse-side effects (11). In this review, the most tested agents corresponded to natural compounds (in 10 out of 19 studies a natural compound were used). This is in line with the afore mentioned argument, as natural compounds have the advantage that are well tolerated by patients and have low number of adverse-side effects.

Ginger is commonly used in traditional Indian and Chinese medicine, and has been suggested to be effective for oral candidiasis and dry mouth (22). As a treatment agent for dry mouth, we found it in two different presentations; capsules (27) and spray (22), but only the presentation as a spray reported to be significant in decreasing dry mouth symptoms and in increasing salivary output (22). Apparently, ginger effects for improving dry mouth symptoms are associated with a local stimulant activity rather than a systemic way of action.

Malic acid is another natural compound which has been attracting attraction during the last years. Malic acid is an organic acid found in fruits, such as pears and apples (33) and act as a salivary stimulant (34). It is commonly combined with fluoride and xylitol to reduce its demineralizing properties. We found two papers that assessed malic acid, whether as a pastille (16) or as a spray (6), and both reported promising results in the improvement of OHRQoL, dry mouth symptoms and salivary flow.

Other natural compounds, such as herbal and plants infusions, lycopene, olive oil, chamomile, and thyme honey, have also been reported as effective for improving dry mouth symptoms (18,19,21,23,28,29).

Five studies evaluated agents that failed to improve oral dryness, which included: chewing gum (26), 4% pilocarpine drops (25), Xerostom® tooth paste and mouthwash (although they did improve OHRQoL) (20), ALTENS (31) and LLLT (32). Although these reports suggest a lack of efficacy of these treatments, care must be taken when interpreting these results. For example, although the study of Duruk and Eser (26) failed to show any effect of chewing gum for the improvement of dry mouth, the effect of chewing gum was tested specifically on patients complaining of dry mouth during hemodialysis, thus is not necessarily extrapolable to other conditions. Also, some of these studies had a reduced sample size (20,25) and high number of dropouts (25,31), thus the lack of significant results could be related to those issues.

We found a large heterogeneity between studies, which made comparisons impossible or extremely difficult. For example, not all studies defined if they were assessing subjective dry mouth (xerostomia) or objective dry mouth (hyposalivation), and others even failed to specify how dry mouth was diagnosed. Also, a variety of outcome measures were assessed between studies using different instruments, including validated and non-validated self-developed questionnaires, scales, and different saliva quantification techniques. This evidences the existing need for developing a core outcome set for reporting dry mouth in clinical trials.

## Conclusions

In the last years, local therapies, particularly the ones based on natural compounds, are the most tested agents in clinical trials for the improvement of dry mouth, reporting good results with no or low number of adverse-side effects. Nevertheless, care must be taken when interpreting these results, as is difficult to compare studies within each other due large heterogeneity in study design and outcomes being measured.
